# Three-dimensional assessment of external apical root resorption after maxillary posterior teeth intrusion with miniscrews in anterior open bite patients

**DOI:** 10.1590/2177-6709.23.6.056-063.oar

**Published:** 2018

**Authors:** Bilal Al-Falahi, Ahmad Mohammad Hafez, Maher Fouda

**Affiliations:** 1 Mansoura University, Faculty of Dentistry, Department of Orthodontics (Mansoura, Egypt).

**Keywords:** Root resorption, Molar intrusion, Miniscrews, CBCT, Anterior open bite.

## Abstract

**Objective:**

The objective of this study was to assess the external apical root resorption (EARR) of the maxillary posterior teeth after intrusion with miniscrews.

**Methods:**

Fifteen patients (13 females and 2 males) with age ranging from 14.5 to 22 years (mean 18.1 ±2.03 years) were selected to participate in this study. All patients presented with anterior open bite of 3 mm or more. An intrusion force of 300 g was applied on each side to intrude the maxillary posterior teeth. Cone beam computed tomography (CBCT) scans were taken pretreatment and post-intrusion and were analyzed to evaluate the EARR.

**Results:**

The maxillary posterior teeth were intruded in average 2.79 ± 0.46 mm (*p*< 0.001) in 5.1 ± 1.3 months, and all examined roots showed statistically significant EARR (*p*< 0.05) with an average of 0.55 mm, except the distobuccal root of the left first permanent molars and both the palatal and buccal roots of left first premolars, which showed no statistically significant changes.

**Conclusions:**

The evaluated teeth presented statistically significant EARR, but clinically, due to the small magnitude, it was not considered significant. Moreover, the CBCT provided a good visualization of all roots in all three planes, and it was effective in detecting minimal degrees of EARR.

## RESUMO

**Objetivo:**

o objetivo desse estudo foi verificar a existência de reabsorção radicular apical externa (RRAE) em dentes posterossuperiores após intrusão ancorada em mini-implantes.

**Métodos:**

quinze pacientes (13 mulheres e 2 homens) com a idade variando entre 14,5 e 22 anos (média de 18,1 ± 2,03 anos) foram selecionados para participar desse estudo. Todos os pacientes possuíam mordida aberta anterior de 3mm ou mais. Uma força de 300 gramas foi aplicada em cada lado para intruir os dentes posterossuperiores. Tomografias computadorizadas de feixe cônico (TCFC), adquiridas antes do tratamento e após a intrusão, foram comparadas para se avaliar a RRAE.

**Resultados:**

os dentes posterossuperiores foram intruídos em média 2,70 ± 0,46 mm (*p*< 0,001) em 5,1 ± 1,3 meses, e todas as raízes examinadas mostraram RRAE estatisticamente significativa (*p*< 0,05), com média de 0,55 mm, exceto pela raiz distovestibular dos primeiros molares permanentes esquerdos, e pelas raízes palatina e vestibular dos primeiros pré-molares esquerdos, que não apresentaram mudanças estatisticamente significativas.

**Conclusões:**

os dentes avaliados apresentaram RRAE estatisticamente significativa, a qual, porém, não foi considerada clinicamente significativa, devido à sua reduzida magnitude. Além disso, a TCFC possibilitou uma boa visualização de todas as raízes nos três planos espaciais, e foi eficaz para detecção de níveis mínimos de RRAE.

## INTRODUCTION

Treatment of anterior open bite has been considered one of the most challenging orthodontic therapies. True molar intrusion is usually needed to correct the skeletal open bite without orthognathic surgery.^1^ ^,^ ^2^

In the last years, skeletal anchorage devices, including miniplates and miniscrews, gained more popularity due to their ability to provide stable anchorage throughout orthodontic treatment.^3-5^ Orthodontic treatment may be the most common cause of EARR in the modern world. The treatment duration, magnitude of applied force, direction of tooth movement, amount of apical displacement, and method of force application are considered the most related risk factors to the EARR.^6^ Furthermore, EARR is one of the most difficult procedure-related adverse events to predict in cases of orthodontic tooth movement (OTM), and may cause permanent loss of the dental structure at the root apex.^7^ EARR is characterized by loss of the external surface layer of cells that protect the tooth roots by the action of clastic cells and hyalinization. Its prevalence is high and it depends on different factors, such as root shape, tooth groups, and measuring techniques.^8^ ^-^ ^10^ However, Roscoe et al^11^ found that there is a positive correlation between the amount of orthodontic force, treatment time and increased EARR.

Several studies^6^ ^,^ ^8^ ^,^ ^9^ ^,^ ^15^ ^-^ ^23^ were conducted to evaluate the EARR of teeth. Research also suggested that individuals with skeletal anterior open bite were at a greater risk of developing EARR during orthodontic treatment than individuals with other types of malocclusion.^21^ Orthodontic intrusion has been described as one of the worst types of OTM in relation to susceptibility to EARR.^22^ Han et al^23^ also concluded that teeth intrusion has four times more chances to cause EARR than extrusion.

Several studies^2^ ^,^ ^24^ ^,^ ^25^ evaluated the EARR after intrusion. However, these studies had used conventional radiographic exams, such as the lateral cephalogram, panoramic and periapical films, to detect the presence of EARR. In addition, these studies were not accurate enough to evaluate the amount of resorption, due to the magnification errors, which might lead to underestimation or overestimation of the amount of root resorption.^26^ ^,^ ^27^ Besides, due to the overlapping of images, not all roots could be examined, such as the palatal roots.

After the scientific and technological developments of medical imaging exams, CBCT was introduced to be a specific diagnostic tool for dentistry.^28,29^ The accuracy of CBCT radiography has already been proved, providing more precise three-dimensional images of the teeth than conventional radiographs.^26^ ^,^ ^30^ ^-^ ^34^

As an examination tool, though, CBCT should be carefully used. The CBCT exposure dose might be 7 to 8 times lower than that of multi-slice CT, and 5 to 6 times higher than that associated with the conventional panoramic radiograph.^35^ ^,^ ^36^

To the best of our knowledge, no study has been performed to evaluate EARR in all posterior teeth in both right and left sides after intrusion with miniscrews in patients with anterior open bite. Therefore, the aim of this study was to evaluate the EARR after intrusion, using measurements based on CBCT.

## MATERIAL AND METHODS

This study was approved by the Ethical Research Committee, Faculty of Dentistry, Mansoura University (Code No: 15020418). 

The sample size was calculated for the difference in maxillary molar length based on a paired samples t-test using the software PS Power and Sample Size Calculations v. 3.1.2 (Department of Biostatistics, Vanderbilt University School of Medicine, Nashville, Tennessee, USA). The mean difference tested for was 0.71 mm. A more liberal standard deviation of the mean difference that was reported by Ari-Demirkaya et al^2^ was used (σ = 0.66 mm), with type I error (alpha significance level) of 0.05 and power of 90%. The estimated sample size was 11 subjects. 

A sample of fifteen patients was selected to participate in this prospective clinical trial. High angle patients with skeletal Class I, II or mild Class III relationship were enrolled in this study. Moderate to severe Class III skeletal relationship patients were excluded, as the molars intrusion would lead to increase the severity of Class III malocclusion. CBCT was used to evaluate 260 roots of 15 non-growing patients (13 female and 2 males), with age ranging from 14.5 to 22 years (mean age of 18.1 ±2.03 years). 

The patients included in this study were selected according to the following criteria:

1. Patients with anterior open bite requiring maxillary posterior teeth intrusion as part of orthodontic treatment.
2. Long-face pattern, with anterior open bite equal to or greater than 3 mm.
3. Healthy adult patients.
4. No previous orthodontic treatment.
5. No evidence of either periodontal problems, gingival problems, or bruxism, at the beginning of orthodontic treatment.
6. No medical problems interfering with orthodontic treatment.

However, patients with a history of trauma and all teeth with endodontically treated roots or with big restoration were excluded from this study.

### Orthodontic treatment progress

Orthodontic bands were cemented on maxillary first and second premolars and first and second permanent molars. Then leveling and alignment were started using sectional wires changed every two weeks, in the following sequence: 0.016-in NiTi, 0.018-in NiTi, 0.016 x 0.022-in NiTi, 0.016 x 0.022-in SS, and 0.017 x 0.025-in SS.

**Figure 1:**
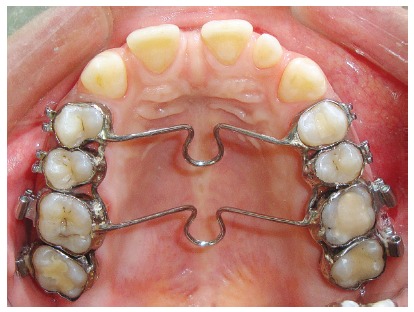
Appliance used to intrude the maxillary posterior teeth.

After leveling and alignment, an impression was taken with the bands on the teeth. Later, the bands were removed of the teeth and reseated on the impression. The impression was delivered to the laboratory for manufacture of the appliance (Fig 1). 

**Figure 2:**
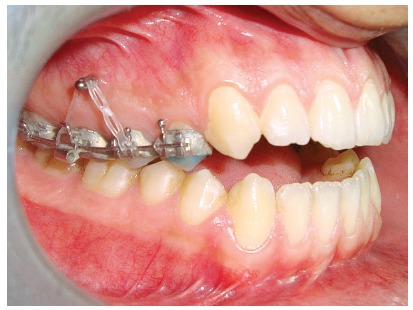
Force application for intrusion.

**Figure 3:**
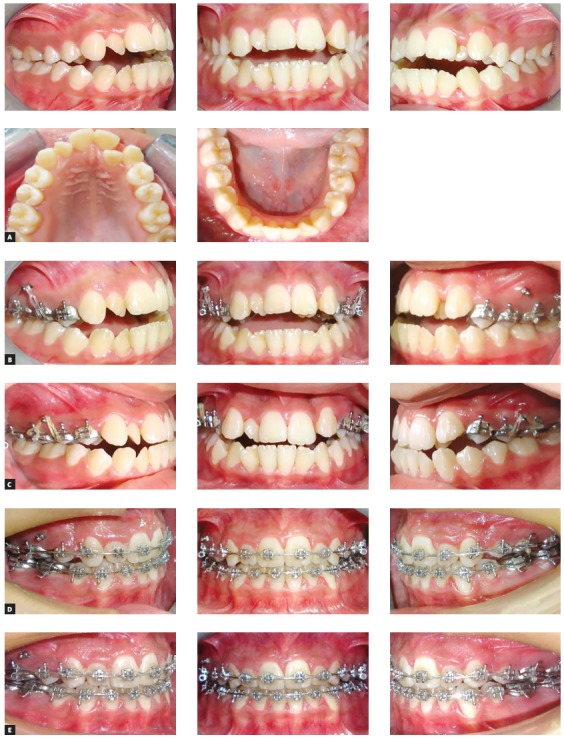
Progress of treatment: A) pretreatment; B) pre-intrusion; C) after maxillary posterior teeth intrusion; D, E) progress of treatment with fixed appliance after intrusion.

The appliance was cemented and a self-drilling titanium alloy mini-screw (1.8 mm in diameter and 8 mm in length) was inserted into the buccal alveolar bone, between the second premolar and first permanent molar on each side. Loading of the miniscrews was initiated two days after insertion and continued until sufficient intrusion had been achieved. An intrusion force of about 300 g was applied on each side by using an elastomeric chain (Memory Power Chain, Ormco™, USA) (Fig 2). Follow-up visits were assigned every two weeks until the required intrusion was obtained. After that, post-intrusion records were taken and analyzed, to evaluate the EARR. However, the orthodontic treatment was continued, with upper and lower fixed appliances, for all cases included in the study (Fig 3).

**Figure 4:**
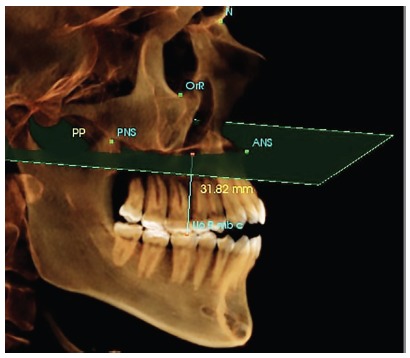
Three-dimensional calculation of the linear distance between the mesio-buccal cusp of maxillary first permanent molar and the palatal plane. The palatal plane was defined as the plane passing through points ANS and PNS, and perpendicular to mid-Sagittal plane, which was constructed during reorientation of the volumetric image.

**Figure 5:**
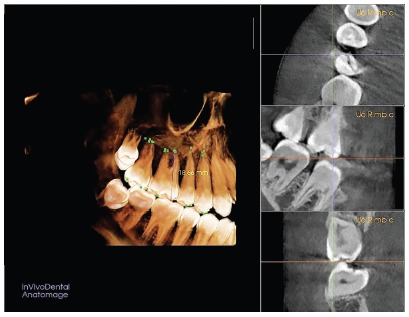
The three-dimensional determination of mesio-buccal cusp landmark of permanent maxillary right first molar (U6 R mbc) on the CBCT volumetric image.

The sectional CBCT scans were obtained at pretreatment (T_1_) and post-intrusion (T_2_), by using i-CAT CBCT machine (Imaging Sciences International, Hatfield, PA). The CBCT machine specifications were as follows: 0.3-mm voxel size, 120 kV, 5 mA, 14.7 seconds exposure time, and 16-cm exposure field, to avoid the exposure to excessive radiation. A three-dimensional (3D) analysis was performed for all CBCT scans, using In Vivo software version 5.01 (Anatomage, San José, USA). After performing the reorientation of the 3D image, the examiner started locating the landmarks. To calculate the amount of molar intrusion performed, difference in the linear distance from the mesio-buccal cusp of maxillary first permanent molar to the palatal plane, between the pretreatment and post-intrusion CBCT records, was measured (Fig 4); while to calculate the amount of root resorption, each cusp tip or root apex was precisely detected in all three planes (sagittal, coronal and axial), for all teeth included in the study. The In Vivo software calculated the maximum linear distance between the two landmarks located by the examiner on both the cusp tip and root apex (Fig 5). The changes between pre and post-intrusion measurements were considered as root resorption. 

The following linear measurements were performed on the 3D volumetric images to all treated patients:

1. Tooth #27 mesiobuccal root: The linear distance between the mesiobuccal cusp and root apex of the mesiobuccal root of the maxillary left second molar.
2. Tooth #27 distobuccal root: The linear distance between the distobuccal cusp and root apex of the distobuccal root of the maxillary left second molar.
3. Tooth #27 palatal root: The linear distance between the palatal cusp and root apex of the palatal root of the maxillary left second molar.
4. Tooth #26 mesiobuccal root: The linear distance between the mesiobuccal cusp and root apex of the mesiobuccal root of the maxillary left first molar.
5. Tooth #26 distobuccal root: The linear distance between the distobuccal cusp and root apex of the distobuccal root of the maxillary left first molar.
6. Tooth #26 palatal root: The linear distance between the palatal cusp and root apex of the palatal root of the maxillary left first molar.
7. Tooth #25 buccal root: The linear distance between the buccal cusp and root apex of the buccal root of the maxillary left second premolar.
8. Tooth #24 palatal root: The linear distance between the palatal cusp and root apex of the palatal root of the maxillary left first premolar.
9. Tooth #24 buccal root: The linear distance between the buccal cusp and root apex of the buccal root of the maxillary left first premolar.
10. Tooth #17 mesiobuccal root: The linear distance between the mesiobuccal cusp and root apex of the mesiobuccal root of the maxillary right second molar.
11. Tooth #17 distobuccal root: The linear distance between the distobuccal cusp and root apex of the distobuccal root of the maxillary right second molar.
12. Tooth #17 palatal root: The linear distance between the palatal cusp and root apex of the palatal root of the maxillary right second molar.
13. Tooth #16 mesiobuccal root: The linear distance between the mesiobuccal cusp and root apex of the mesiobuccal root of the maxillary right first molar.
14. Tooth #16 distobuccal root: The linear distance between the distobuccal cusp and root apex of the distobuccal root of the maxillary right first molar.
15. Tooth #16 palatal root: The linear distance between the palatal cusp and root apex of the palatal root of the maxillary right first molar.
16. Tooth #15 buccal root: The linear distance between the buccal cusp and root apex of the buccal root of the maxillary right second premolar.
17. Tooth #14 palatal root: The linear distance between the palatal cusp and root apex of the palatal root of the maxillary right first premolar.
18. Tooth #14 buccal root: The linear distance between the buccal cusp and root apex of the buccal root of the maxillary right first premolar.

### Methods error 

The measurements of the present study were performed by one orthodontist, as the software needs a skilled operator to locate the landmarks. To assess the reliability of the method, the intraclass correlation coefficient (ICC) analysis was used. According to Roberts and Richmond,^37^ reliability is excellent if ICC value is higher than 0.75; acceptable if it is between 0.4 and 0.75; and low if the ICC is smaller than 0.4. 

In the present study, the ICC showed excellent intra-examiner reliability. The ICC for linear measurements showed an average of 92.6%, with a range from 0.827 to 0.995, and the used method presented high reproducibility. 

### Statistical analysis

The statistical analysis was performed with the software Statistical Package for the Social Sciences v. 24.0 (SPSS, Chicago, IL, USA). Data were explored for normality using Shapiro-Wilk test, showing normal distribution. A descriptive statistical analysis was used to present the data as mean and standard deviation (SD). Paired sample *t*-test was used to evaluate the significance of the difference in the pre- and post-intrusion data. 

## RESULTS

The maxillary posterior teeth were truly intruded, with an average of 2.79 ± 0.46 mm. The mean time for maxillary posterior teeth intrusion was 5.1 ±1.3 months. Results of the present study revealed that all examined roots showed statistically significant (*p*< 0.05) EARR, which ranged from 0.34 to 0.74 mm, between pre- and post-intrusion measurements (Table 1).

**Table 1:** Pre- and post-intrusion changes and significance.

Variables	Pre-intrusion		Post-intrusion		Difference		P-value	Significance^a^
	Mean	SD	Mean	SD	Mean	SD		
Maxillary second molars distobuccal root	18.60	1.30	18.07	1.28	0.53	0.40	0.004	**
Maxillary second molars mesiobuccal root	19.87	1.29	19.23	1.35	0.34	0.40	0.033	*
Maxillary second molars palatal root	20.32	1.43	19.57	1.71	0.74	0.63	0.008	**
Maxillary first molars mesiobuccal root	19.49	0.84	18.87	0.95	0.61	0.43	0.003	**
Maxillary first molars distobuccal root	18.71	0.92	18.09	1.00	0.62	0.45	0.003	**
Maxillary first molars palatal root	20.94	1.29	20.24	1.47	0.70	0.50	0.003	**
Maxillary second premolars buccal root	20.93	1.12	20.44	1.23	0.48	0.53	0.026	*
Maxillary first premolars buccal root	21.15	1.38	20.58	1.13	0.57	0.56	0.017	*
Maxillary first premolars palatal root	19.89	1.03	19.37	1.08	0.52	0.54	0.020	*

^a^ NS= non-significant; ? *p* < 0.05; ** *p* < 0.01; *** *p* < 0.001.

## DISCUSSION

The aim of the present study was to evaluate the EARR of maxillary posterior teeth after intrusion, by using CBCT. According to the literatures, there is no safe tooth movement with regard to EARR. Because intrusion is probably the most detrimental to the roots involved,^25^ ^,^ ^38^ this study attempted to evaluate the effects on root structure caused by intrusion of posterior teeth with mini-implants.

The identification of the landmarks is considered the main source of error inherent in the measuring procedure. The conventional two-dimensional imaging methods show a high frequency and overestimate EARR after orthodontic treatment;^12^ ^,^ ^14^ ^-^ ^16^ however, CBCT images provided a more accurate analysis of treatment results. By comparing the accuracy of CBCT to that of periapical radiographs with regard to detection of EARR, several studies showed that the three-dimensional method was more effective and reliable.^10^ ^,^ ^29^ ^-^ ^32^ ^,^ ^36^

Although a number of studies have already evaluated EARR using CBCT images, the present study allowed a total view of resorption (possible resorption in all roots submitted to orthodontic forces). In the present study, a specific CBCT software was used to obtain accurate linear measurements of teeth in millimeters. Crowns without metal restorations or fractures were included in the study to ensure good visualization of the images and to avoid image artifacts. Three-dimensional tracing of volumetric CBCT images allows an accurate detection of a specific landmark in all three planes, and minimizes limitations inherent to conventional two-dimensional radiographs, such as lack of standardized radiographic technique and overlapping of teeth. However, from the insignificantly small method error, it can be concluded that the CBCT images and the software used in this study have the ability to provide a clear 3D image that shows the small details of different anatomical landmarks of the teeth, thus minimizing the possible errors during measuring procedures.

In this study, the maxillary posterior teeth were effectively intruded a mean of 2.79 ± 0.46 mm in 5.1 ± 1.3 months. Results showed that all intruded teeth presented with statistically significant EARR, with a mean of 0.55 mm, ranging from 0.34 to 0.74 mm. This result is in harmony with the findings of Heravi et al,^24^ of 0.3-0.4 mm of root length loss, and Li et al,^26^ who reported statistically significant EARR. On the other hand, there were less EARR in this study than in the one reported by Dermaut and De Munck^25^ (2.5mm), as they had evaluated the EARR in the maxillary incisors, where there was more occurrence of EARR with continuous forces.^14^ ^,^ ^15^ Acar et al^39^ indicated that the application of intermittent force results in less EARR than does the application of continuous force. Moreover, Ari-Demirkaya et al^2^ reported higher EARR after intrusion of maxillary first molars (0.8 mm). This difference may be due to the longer duration of treatment (20 months), in comparison with 5.1 months in this study. In addition, they used panoramic radiographs to assess EARR, which can overestimate resorption amount.^12^ On the contrary, there were less EARR in this study than in the one reported by Castro et al.^10^ The difference in the type of tooth movements,^23^ ^,^ ^40^ as they studied the EARR in patients with crowding treated with nonextraction strategy, might explain the different results.

The correlation between EARR and orthodontic treatment has been thoroughly studied, but the comparison of the results is difficult as a result of heterogeneity among different studies, regarding techniques of treatment, radiographic evaluation criteria, and imaging methods.^12^ ^,^ ^16^ ^,^ ^17^ ^,^ ^19^ Although the results of the present study were statistically significant, it is still considered clinically non-significant. The relatively small amount of EARR may be due to the optimal level of force used (300 g), as the high force levels correlate to the EARR, in addition to the relatively small intrusion period (5.1 ± 1.3 months).^11^ ^,^ ^13^ ^,^ ^33^

This study evaluated the EARR of all maxillary posterior teeth in both sides after orthodontic intrusion, by using a 3D analysis software that helped to analyze a volumetric image by which the anatomical landmarks were located directly on the 3D image. So, all parts of the tooth structures were visualized without overlapping, in a very clear and accurate image. 

## CONCLUSIONS

» All evaluated teeth had statistically significant EARR; but, because of its small magnitude, it should be considered as clinically irrelevant.
» The CBCT provided a good visualization of all examined roots in all three planes of space, specially the palatal roots of posterior teeth, without overlapping or magnification errors.
